# Chloride as a Partial Nitrate Substitute in Hydroponics: Effects on Purslane Yield and Quality

**DOI:** 10.3390/plants14142160

**Published:** 2025-07-13

**Authors:** George P. Spyrou, Ioannis Karavidas, Theodora Ntanasi, Sofia Marka, Evangelos Giannothanasis, Gholamreza Gohari, Enrica Allevato, Leo Sabatino, Dimitrios Savvas, Georgia Ntatsi

**Affiliations:** 1 Laboratory of Vegetable Production, Department of Crop Science, Agricultural University of Athens, Iera Odos 75, 11855 Athens, Greece; gspyrou@aua.gr (G.P.S.); karavidas@aua.gr (I.K.); ntanasi@aua.gr (T.N.); giannothanasis@aua.gr (E.G.); dsavvas@aua.gr (D.S.); 2 Laboratory of Molecular Biology, Department of Biotechnology, Agricultural University of Athens, 11855 Athens, Greece; smarka@aua.gr; 3 Department of Horticultural Science, Faculty of Agriculture, University of Maragheh, Maragheh 83111-55181, Iran; gohari.gh@maragheh.ac.ir; 4 Department of Environmental and Prevention Sciences (DiSAP), University of Ferrara, Via Borsari, 46, 44121 Ferrara, Italy; enrica.allevato@unife.it; 5 Dipartimento Scienze Agrarie, Alimentari e Forestali, University of Palermo viale delle Scienze, Ed. 5, 90128 Palermo, Italy; leo.sabatino@unipa.it

**Keywords:** ammonium nitrogen, chloride, floating system, nitrate nitrogen, plant nutrition, *Portulaca oleracea* L., uptake concentrations

## Abstract

This study examined the effects of both nitrogen (N) rate and form on the growth, nutrient uptake, and quality parameters of hydroponically grown purslane (*Portulaca oleracea* L.) during a spring cultivation cycle. Purslane was cultivated in a floating hydroponic system under either adequate or limiting N conditions. More specifically, under adequate N conditions, plants were supplied with NS where ammonium nitrogen (NH_4_-N) accounted for either 7% (Nr7) or 14% (Nr14) of the total-N. The limiting N conditions were achieved through the application of either an NS where 30% of N inputs were compensated with Cl (N30), or an NS where 50% of N inputs were balanced by elevating Cl and S by 30% and 20%, respectively (N50). The results demonstrated that mild N stress enhanced the quality characteristics of purslane without significant yield losses. However, further and more severe N restrictions in the NS resulted in significant yield losses without improving product quality. The highest yield reduction (20%) occurred under high NH_4_-N supply (Nr14), compared to Nr7-treated plants, which was strongly associated with impaired N assimilation and reduced biomass production. Both N-limiting treatments (N30 and N50) effectively reduced nitrate accumulation in edible tissues by 10% compared to plants grown under adequate N supply (Nr7 and Nr14); however, nitrate levels remained relatively high across all treatments, even though the environmental conditions of the experiment favored nitrate reduction. All applied N regimes and compensation strategies improved the antioxidant and flavonoid content, with the highest antioxidant activity observed in plants grown under high NH_4_-N application, indirectly revealing the susceptibility of purslane to NH_4_-N-rich conditions. Overall, the form and rate of N supply significantly influenced both plant performance and biochemical quality. Partial replacement of N with Cl (N30) emerged as the most promising strategy, benefiting quality traits and effectively reducing nitrate content without significantly compromising yield.

## 1. Introduction

Recent advancements in botanical research have resulted in the expansion of the global database of plant species, revealing their vast potential for human use. It is currently estimated that at least 7039 plant species are suitable for human consumption, with additional applications in other sectors such as medicine, construction, and environmental and energy-related industries [1]. Despite this huge number of species, the Food and Agriculture Organization (FAO) has stated that only 50 species are actively cultivated worldwide, with just 17 species accounting for 90% of the global food supply [2]. This situation of heavily relying on a limited number of crops, coupled with the predicted escalation of the global population from approximately 8 billion to 9.7 billion individuals by the year 2050, raises profound concerns about ensuring food security and availability, while preserving biodiversity [3].

Addressing these challenges necessitates both shifting towards novel small-scale and local cultivation systems [4,5,6] and exploiting and preserving novel and regionally adapted plant species able to enhance food diversity and agricultural sustainability [7,8]. Such plant species, often overlooked by global markets, are characterized as “underutilized” or “neglected” species (NUS). Both terms refer to species that are naturally grown in a specific region and traditionally cultivated by local farmers due to their high nutritional value but lack widespread commercial adoption [9]. *Portulaca oleracea* L., commonly referred to as purslane, is a prime example of NUS. Purslane, primarily originating from the Mediterranean region but distributed globally, is considered a highly nutritious leafy vegetable [10,11]. This is ascribed to its significant amounts of potassium (K), calcium (Ca), magnesium (Mg), and iron (Fe), making it a valuable source of these important nutrients [12]. The high concentrations of linolenic acid (α- & γ-), beta-carotene, and ascorbic acid found in purslane exceed the levels found in major commonly cultivated leafy vegetables [13,14], while the notable abundance of cholesterol-free omega-3 fatty acids constitutes this crop an alternative to fish oils, thereby characterizing it a “niche” product [14,15,16].

Apart from its high nutritional properties, purslane has been characterized as a climate-resilient crop due to its ability to thrive in challenging conditions, making it a valuable option for advancing sustainable agriculture in the face of climate change [17]. A considerable number of studies have been conducted with the aim of evaluating the level of resilience of purslane to abiotic stresses. More specifically, purslane showed a notable adaptation to mild salinity stress (50 mM), maintaining growth performance, net photosynthetic rate, and macronutrient concentration (Na, K, Mg, and Ca) [18]. According to [19], purslane has been shown to possess significant resilience to drought-related stress, with the capacity to trigger a range of physiological responses that facilitate more expeditious recovery under rehydration. Furthermore, research based on combined stresses has highlighted the ability of purslane to combat combined abiotic stresses through the activation of physiological and metabolic pathways [20,21,22]. Additionally, purslane has been recognized as a phytoremediation species, a term given to a plant that is capable of accumulating significant quantities of heavy metals in contaminated habitats. Indeed, purslane has demonstrated noteworthy phytoremediation efficiency with respect to lead (Pb), cadmium (Cd), and nickel (Ni), as well as plant tolerance to mild concentrations of the above-mentioned elements [23,24,25]. All the above constitute this species’ low input and cost-effectiveness, strengthening its potential to be introduced into sustainable and highly productive cultivation systems.

Despite its resilience, purslane is highly responsive to fertilization practices, especially those concerning N application, an essential macronutrient that determines yields in cropping systems. Specifically, N fertilization is critical for optimizing both yield and quality of purslane in both soil and soilless systems; however, excessive N fertilization may not have a beneficial effect on yield [26,27,28,29] or even hinder plant growth and quality performance [30,31]. Moreover, excessive N application can result in increased nitrate abundance, with major environmental burden via groundwater contamination and greenhouse gas emissions, contributing to eutrophication, soil degradation, and biodiversity loss [32,33]. Ammonium fertilization, as an alternative nitrogen management strategy, has been adopted to reduce nitrate accumulation in sustainable agriculture [34,35]. However, its overapplication can also disrupt ecological balance due to its adverse effects, including soil acidification, ammonia volatilization, and ammonia toxicity, which can negatively impact crop performance, soil microbial communities, and aquatic organisms [36,37,38,39,40]. Consequently, the substantial losses of anthropogenic nitrogen pose a risk to human health and contribute to the triple planetary crisis of climate change, pollution, and biodiversity loss [41]. Therefore, optimal N nutrition can not only increase food quality and consumer safety but also prevent N leaching to soil and water bodies and atmospheric N losses.

A major concern regarding the nutritional properties of leafy vegetables is their tendency to accumulate excessive amounts of nitrate, resulting in high concentrations in the edible part of the plant [42,43]. There are several proposed strategies to overcome this issue, such as reduced N supply or partial substitution of NO_3_-N with NH_4_-N. However, excessive ammonium supply is associated with ammonia toxicity, which can disrupt cytosolic pH regulation, nutrient uptake, photosynthetic efficiency, plant morphology, and nitrogen assimilation processes [44,45,46], while reduced N application may result in reduced growth. Moreover, replacing NO_3_-N with chloride (Cl) has been efficiently proven effective in enhancing N use efficiency (NUE) and suppressing nitrate accumulation in leafy vegetables without compromising yield in soilless cultivation systems [47,48,49].

Taking all the above into consideration, an experiment was designed with the aim of suggesting nitrogen-related fertilization strategies that aim to reduce nitrate levels in the plant tissue of hydroponically grown purslane without compromising crop performance and nutritional value. To attain this goal, the three different strategies mentioned above (i.e., reduced N supply, substitution of NO_3_-N with either NH_4_-N or Cl) were tested to reveal the most effective one for mitigating nitrate accumulation without adversely affecting yield or nutritional quality.

## 2. Results

### 2.1. Yield and Growth Parameters

According to Table 1, plant fresh weight was significantly reduced in both severe-N (N50) and Nr14 treatments compared to Nr7-treated plants, by 10% and 20%, respectively. In N50-treated plants, the yield gap was accompanied by a 12% decrease in leaf area, while the total leaf number per plant did not significantly differ from that of Nr7. In contrast, the yield gap in Nr14-treated plants was due to both a 17% reduction in leaf number and a 20% decrease in leaf area compared to Nr7. In addition, the negative effects of Nr14 were observed in both shoot and root dry weight, indicating a detrimental impact on purslane biomass characteristics. Nr14 treatment also led to an increase in aboveground biomass dry matter content, a common stress indicator, which was approximately 8% higher than in Nr7. However, plants treated with mild N stress (N30) did not exhibit any negative effects on growth, as none of the evaluated biomass characteristics were affected under this condition.

### 2.2. Plant Nutrient Profile

Plants treated with Nr7 and N30 showed higher levels of reduced N (Nred) (Table 2). In contrast, N50 and Nr14 treatments resulted in Nred reductions of 3.5% and 11%, respectively, compared to Nr7. Both nitrogen deficit treatments (N30 and N50) resulted in a 13% reduction in mineral N (Nmin) levels compared to plants receiving an adequate N supply (Nr7 and Nr14). In addition, all alternative N fertilization strategies resulted in a reduction of about 7% in the total N content of purslane plant tissues. With regard to the ratio of reduced N to total N, plants receiving limited N inputs (N30 and N50) showed higher values, whereas plants receiving high NH_4_-N (Nr14) inputs showed lower values. Additionally, under sufficient N supply, Nr14 plants recorded a reduced Nred/Ntotal ratio by 4% compared to Nr7-treated plants. Finally, both nitrogen deficit treatments resulted in a 10% decrease in nitrate (NO_3_) accumulation in fresh purslane leaves when compared to plants grown under adequate N supply (Nr7 and Nr14).

The different N fertilization practices strongly affected both the macro- and micronutrient profile of purslane shoots (Table 3). As for macronutrients, high NH4-N application favored P and Na contents compared to the control and mild N treatments. Both deficit N treatments (N30 and N50) resulted in higher Cl content, a reasonable effect due to the Cl supplementation in NS. Concerning the micronutrient content, Nr7- and N30-treated plants recorded higher values for Fe concentration in the purslane shoot. However, the Nr7-treated plants also recorded the lowest Cu concentration. Finally, the different N treatments had no effect on the levels of K, Ca, Mg, B, Zn, and Mn in purslane shoots. Regardless of the fertilization management, plants recorded a considerably high K concentration, more than twice of N content, and a too low Ca concentration. In contrast to the nutrient levels in the shoots, the different N management practices did not have a significant effect on the nutrient concentration in the roots of purslane (Table 4). In particular, the only significant variation was observed in Cu content, where higher values were recorded in Nr14-treated plants.

### 2.3. Uptake Concentrations

The two different methods employed to determine nutrient requirements for hydroponic production of purslane had a great impact on plant macronutrient uptake concentrations (Table 5). The UCs estimated through plant tissue nutrient analysis (UC-DB) were significantly lower than those estimated through NS analysis (UC-NS). Specifically, UC-DB was lower than UC-NS for N, P, K, Mg, and Na by 2.6%, 12.8%, 8.9%, 9.3%, and 12.3%, respectively. Greater variation was observed in UC of Ca, where the UC-DB value represented approximately 6% of that of UC-NS. Irrespective of the different methods employed, the different N managements affected only the UCs of N and Cl. Particularly, Nr7-treated plants recorded the highest UC (14.39 mM) of N, while in plants treated with either Nr14 and N30 or N50, the respective values were limited by 8% and 14% compared to Nr7-treated plants, respectively. The interactions between the two factors revealed that the UC of N in Νr14-treated plants was equal to that of Nr7 under the NS method (UC-NS). Under the DB method, the UC (UC-DB) of N in Nr14-treated plants decreased by 14.2% compared to Nr7 and ranged at the same levels as that recorded by the plants in deficit N treatments N30 and N50. Concerning Cl UC, plants grown under both deficit N applications recorded twice the values compared to those of the N adequate supply.

Contrary to the macronutrient UCs, neither the different N management nor the interactions between the experimental factors resulted in significant variations (Table 6). However, a similar trend was observed for micronutrient UCs for the effect of the different methods used. Specifically, the UC-NS method resulted in higher UCs for all micronutrients except Cu. The UC-DB method resulted in lower UCs for B and Zn by 7.5% and 12.5%, respectively, while the respective decrease in UCs for Fe and Mn was 17% and 20%, respectively.

### 2.4. Nitrogen Use Efficiency (NUE)

**Figure 1:**
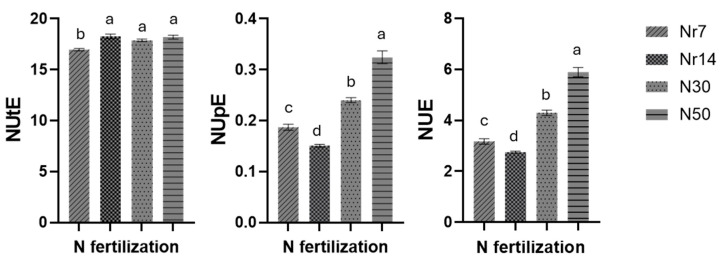
Impact of the different N managements on NUtE (1A), NUpE (1B), and NUE (1C). Error bars extending above and below each point represent the standard error of the mean (SE). Different letters indicate statistically significant differences according to the Duncan multiple range test at *p* < 0.05. Nr7 denotes for 7% constitution of nitrate nitrogen (NO_3_-N) with ammonium nitrogen (NH_4_-N), Nr14 denotes for 14% constitution of nitrate nitrogen (NO_3_-N) with ammonium nitrogen (NH_4_-N), N30 denotes for compensating 30% of N inputs with Cl, and N50 denotes for compensating 50% of N inputs in the nutrient solution (NS) with 30% Cl and 20% S.

The impact of diverse nitrogen management treatments on the three nitrogen indices (NUtE, NUpE, and NUE) was investigated. In particular, the lowest NUtE value was observed in plants cultivated in Nr7 NS, while the NUpE value exhibited variation depending on the N treatments, attaining its maximum value under N50 and its minimum value under Nr14 (Figure 1). The same pattern was also observed in the NUE index, where N50 increased NUE values, followed by N30-treated plants.

### 2.5. Biochemical Profile

**Figure 2:**
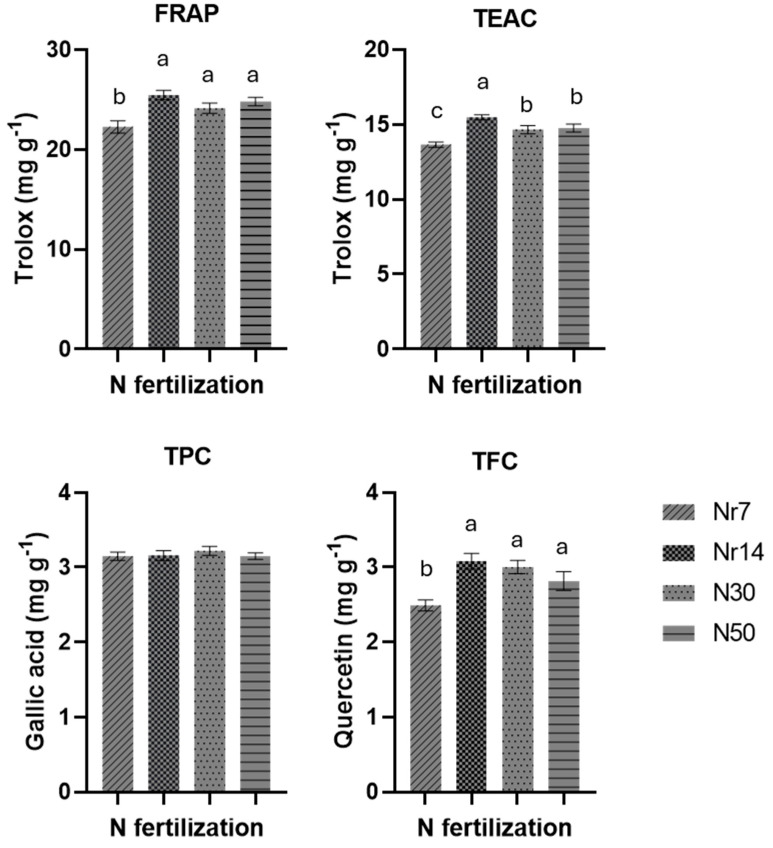
Impact of the N management treatments on plant biomass antioxidant activity (FRAP and TEAC) and total phenolic (TPC) and flavonoid content (TFC). Error bars extending above and below each point represent the standard error of the mean (SE). Different letters indicate statistically significant differences according to the Duncan multiple range test at *p* < 0.05. Nr7 denotes for 7% constitution of nitrate nitrogen (NO_3_-N) with ammonium nitrogen (NH_4_-N), Nr14 denotes for 14% constitution of nitrate nitrogen (NO_3_-N) with ammonium nitrogen (NH_4_-N), N30 denotes for compensating 30% of N inputs with Cl, and N50 denotes for compensating 50% of N inputs in the nutrient solution (NS) with 30% Cl and 20% S.

In this study, to determine the antioxidant properties of purslane methanolic extracts, two assays were performed, the Trolox equivalent antioxidant capacity (TEAC) and ferric reducing antioxidant power (FRAP). Both methods revealed that Νr7-treated plants recorded the lowest antioxidant capacity, while the supply of NS rich in NH4 (Nr14) produced purslane plants with the highest antioxidant potential (Figure 2). Additionally, all alternatives to Nr7 fertilization management equally enhanced the plant total flavonoid content. Finally, total phenolic content in purslane extracts seems not to be affected by the applied N fertilization practices.

## 3. Discussion

Purslane (*Portulaca oleracea*), while exhibiting notable tolerance and adaptability to adverse environmental conditions and suboptimal soil quality, is highly responsive to N fertilization practices. As a key macronutrient, N plays a crucial role in optimizing both yield and quality of purslane grown under either soil or soilless culture systems. Although N fertilization is essential for purslane production, several studies have demonstrated that over-fertilization may have either no significant yield benefits [26,27,28,29] or even detrimental impacts on crop performance [30,31]. On the other hand, [50] reported that purslane biomass was not significantly influenced by nitrogen concentrations ranging from 1 to 7.5 mM under hydroponic conditions. However, these levels are considered suboptimal for the hydroponic cultivation of leafy greens [51], which may have affected the observed response. This study supports the notion that purslane demonstrates moderate sensitivity to external N levels. In particular, yield losses (by 10%) were observed when plants were exposed to a 50% reduction in N application (N50), a response that is attributed to both the restricted leaf number and total leaf area. On the contrary, plants that received 70% N (N30) input compared to the control did not experience any negative effect on either plant yield or plant biomass characteristics.

Beyond the importance of nitrogen (N) application rates, the source of Ν appears to play an even more critical role in hydroponic purslane production. According to the outcomes of this study, elevating the Nr ratio (Nr14), while maintaining high total-N inputs, resulted in the most detrimental effects on plant biomass characteristics. This suggests that the form of N supplied may have a greater impact on plant performance than total-N quantity, in agreement with prior research on hydroponically grown leafy vegetables [52,53]. Purslane’s negative responses in terms of growth under elevated NH_4_-N levels were also observed in numerous studies [54,55,56], where purslane was supplied with an NS where Nr ranged above 0.25. However, in these studies, the NH_4_-N/Total-N ratios investigated surpass the generally recommended ratios of 0.10 to 0.15 for optimal hydroponic vegetable production in closed-loop systems [51,52,53,57,58,59]. Within this recommended range, in a study, it has been reported that ratios above 0.1 during spring cultivation periods can inhibit the growth of purslane grown in NFT systems [60]. In the present study, the Nred/Total-N ratio and Total-N content in purslane plant tissues were greater in control-treated plants than that of plants treated with high ammonia supply, indicating that excess ammonia application restricted both N uptake and plant N assimilation rate. Excessive ammonium supply is associated with ammonia toxicity, which can disrupt cytosolic pH regulation, nutrient uptake, photosynthetic efficiency, plant morphology, and nitrogen assimilation processes [44,45,46]. In addition to plant N content, the different N management strategies also influenced the concentrations of P, Na, Cu, and Fe in the aboveground biomass of purslane. In particular, higher Nr enhanced significantly the P concentration of the plants. Similar reports were also observed in the study of Savvas et al. [61] where high Nr ratio benefited P uptake, a trend that was mainly attributed to the beneficial acidifying effects of NH_4_-N application in the root-zone pH. Comparable effects of the N managements on P content were also observed in Na and Cu content in plants. Provided that higher P, Na, and Cu concentrations were recorded in plants that suffered the most detrimental effects on biomass production due to the different N managements, and that the N managements did not influence the uptake rate of these nutrients, we can conclude that these higher concentrations are mainly ascribed to the accumulation of these nutrients at the dry aboveground biomass of plants. In the case of Fe concentration, higher levels were recorded in Nr7 and N30 plants, which were also associated with elevated Nred levels. These outcomes are in line with earlier findings that link efficient N uptake and assimilation with improved Fe acquisition and transport in plants [62,63].

In the current study, both UC estimation methods and N fertilization practices contributed significantly to the variation in plant N uptake concentration. Specifically, as the N supply increased the N uptake rate by the plants was also increased, which agrees with previous studies reporting that increased N supply results in rich N uptake by plants [64]. Regarding the different estimation methods, a significant gap in N uptake concentration was observed only in plants grown under high Nr. Specifically, the NS-based method that considers the whole N losses from NS during the whole cropping period recorded higher N UC values compared to UC-DB method that reflects the total amount of N utilized by plants. Considering that this significant variation was observed only in high-NH4-treated plants, this gap is presumably attributed to the losses of N from the NS via denitrification, a trend that has previously been reported by Daum and Schenk [65] and Xaxiri et al. [66]. Estimates by Karlowsky et al. [67] and Lin et al. [68] suggested that denitrification can account for approximately 12% of the total N supplied in hydroponic systems. Furthermore, several studies have highlighted the role of root zone pH in influencing nitrification and denitrification rates. Šimek and Cooper [69] reported that pH levels above 7 favor denitrification, while Daum and Schenk [70] demonstrated that maintaining root zone pH between 5.5 and 6.2 can effectively limit N losses below 10%.

Beyond N, the use of different estimation methods resulted in notable discrepancies in the UC values for nearly all nutrients evaluated in this study. For K, Na, Cl, and Mg, the observed differences between the NS- and DB-based estimation methods remained within 10%, a variation considered acceptable given the methodological differences in nutrient quantification across solution and biomass matrices. Similar findings were also reported by other studies [47,66,71], where no substantial or significant variations were observed in assessing UC values through NS and DB calculations. The minor differences in P and Ca UCs, amounting to approximately 0.15 and 0.4 mM, respectively, are mainly attributed to the precipitation of these nutrients due to the formation of insoluble salts, a common occurrence in soilless culture that is further exacerbated as pH in the rootzone increases [72,73]. Both UC determination methods indicated a poor uptake capacity for Ca. Interestingly, regardless of the N management strategy applied, purslane exhibited an unusually high accumulation of K and a remarkably low accumulation of Ca, deviating from the typical mineral composition reported in prior studies [14,74,75]. The diminished Ca uptake can primarily be attributed to both the K/Ca ratio in the root zone and, more significantly, the exceptionally rapid growth of the plant [51,76,77]. In this study, Ca concentrations in the dry aboveground biomass averaged approximately 0.3 mg/g, which is notably below the 1.0 mg/g threshold commonly associated with the onset of tip burn in lettuce [51]. Nonetheless, no visual symptoms of Ca deficiency or tip burn were observed in purslane throughout the cropping period.

In addition to Ca and P, the availability of trace elements in hydroponic systems is also influenced by the chemical characteristics of the nutrient solution. Specifically, trace elements, including Fe, Mn, Zn, and Cu, can form insoluble bases, limiting their availability due to precipitation, particularly under elevated pH conditions in the nutrient solution, restricting also their bioavailability for plant uptake [66,73,78,79]. Except for the varying nutrient availability due to the chemical properties of the nutrient solution, discrepancies observed in micronutrient UC may also be attributed to methodological differences in nutrient quantification. Specifically, the recovery of micronutrients such as Fe and Mn from dried plant tissue can be significantly influenced by the digestion method, particularly the temperature and retention time of the ashing process, thereby introducing variability in measured concentrations [80].

In terms of product quality, nitrogen (N) limitation has been previously associated with improved biochemical profiles in leafy vegetables. Prior studies have demonstrated that reduced N availability can enhance secondary metabolite synthesis, including antioxidant compounds and flavonoids [81,82,83,84]. The result of the study indicates that both deficit N treatments (N30 and N50) primarily enhanced antioxidant activity and flavonoid content without significantly affecting total phenolic content. This pattern aligns with the findings of de Jesus et al. [26], who reported similar responses in purslane cultivated under varying N regimes. Both antioxidant assays revealed that the higher antioxidant performance was reported in plants treated with higher Nr, indicating indirectly that the plants experienced a higher oxidative stress. This interpretation is further supported by the observed detrimental effects of high Nr on both nitrogen assimilation rate and plant vigor.

Regarding nitrate accumulation in edible plant tissues, both Nr7 and Nr14 and NO_3_-N partial substitution by Cl comprise fertilization strategies to either monitor or maintain nitrate levels within acceptable thresholds according to EU (No1258/2011) regulation [18,47,61,85,86,87]. The replacement of NO_3_-N inputs by Cl has been efficiently adapted as a strategy to reduce nitrate accumulation in hydroponically grown leafy vegetables without compromising yield [47,48,49]. In the current study, however, only the N-deficit treatments suppressed nitrate accumulation, while, irrespective of the N management, the nitrates ranged in considerably too high levels, provided that the trial was conducted during the spring cultivation period with ambient light conditions. Similar trends were also reported by Nicola et al. [88], where nitrate accumulation was suppressed by either limiting N supply or elevating the extreme Nr (>0.4) ratio in hydroponic purslane.

## 4. Materials and Methods

### 4.1. Plant Material, Growth Conditions, and Experimental Setup

**Figure 3:**
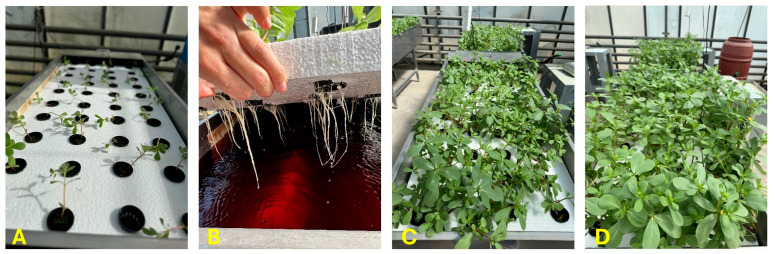
The crop progresses from the stage of transplanting (**A**), to 5 days after transplanting (**B**), 10 days after transplanting (**C**) and finally to the stage of harvest stage ((**D**), which occurred 14 days after transplanting).

The experiment was conducted at the glasshouse facilities of the Laboratory of Vegetable Production at the Agricultural University of Athens (37°58′57.8″ N, 23°42′14.3″ E). On 14/03/2024, purslane seeds (Primasem SA, Athens, Greece) were sown on rockwool trays (AO Plug, Grodan, Roermond, The Netherlands), and at the stage of first 2–3 true leaves (approximately X days after sowing), the seedlings were transplanted on 16 floating tanks (Figure 3). Each tank measured 30 cm deep, 55 cm wide, and 180 cm long, constructed with stainless steel (IntelAgro, Thermi, Greece) and contained 180 L of nutrient solution (NS). Tanks accommodated 40 plants each, resulting in a plant density of 40 plants m^−2^. To ensure that the dissolved oxygen (O_2_) concentration remained above 6 mg L^−1^, each tank was equipped with an air stone, while the recirculation of the nutrient solution was facilitated by an immersion pump.

In this study, the impact of diverse nitrogen (N) fertigation strategies on hydroponic purslane (Portulaca oleracea) cultivation was examined. Specifically, the plants were cultivated under two distinct nitrogen (N) regimes. Plants that grown under adequate N conditions were supplied with an NS, where NH4-N levels represented either 7% (Nr7 = 0.07, Control) or 14% (Nr14 = 0.14, High NH_4_-N) of total N content. In deficit N conditions, plants were either supplied with an NS where 30% of NO_3_-N inputs were compensated with Cl (Mild-N stress: N30), or an NS where 50% of NO_3_-N inputs were compensated by elevating Cl and S by 30% and 20% (Severe N stress: N50). Each treatment was replicated four times, with each floating tank representing one treatment replication. The NSs among the treatments were isosmotic, and their composition, reported on Table 7, was determined via NUTRISENSE (https://nutrisense.online/, accessed on 1 April 2024), an online Decision Support System [89]. The electrical conductivity (EC) and pH in the NSs were recorded in daily intervals and are shown in Figures S1 and S2, respectively.

### 4.2. NS Sampling

The cropping period from the transplanting (2 April 2024) to harvest (16 April 2024) lasted only 15 days (Figure 3). NS samples were collected from each floating tank on the start and end dates of the cultivation period. The samples were analyzed for their nutrient concentration immediately after sampling. In addition to NS sampling, at the harvest date, the volume of NS per tank that was consumed by plants during the whole growth cycle was recorded.

### 4.3. Plant Tissue Sampling

The harvesting of the plants occurred at the point of first flower emergence. During the harvesting process, eight fully grown, well-developed plants were randomly selected from each treatment to ensure uniformity. The fresh weight (g/plant), leaf number (N/plant), and leaf area (cm^2^/plant) of these plants were meticulously recorded, and the values were averaged to obtain the mean per treatment replication. For the determination of dry matter content and nutrient composition, the eight sampled plants were divided into two subsamples per tank. In addition, the roots of all 40 plants per tank were harvested. Both fresh shoot and root samples were washed with distilled water and then placed in an oven at 65 °C (STF-N 400, FALC Instruments S.L.R, Treviglio, Italy) for drying up to a constant weight. Following the determination of the dry weight of the samples, the dried material was pulverized using an MF 10 Microfine grinder (IKA Werke, Staufen, Germany) and stored for the subsequent determination of their nutrient content. In addition, the remaining 32 plants per tank were harvested, and their fresh weight was recorded to estimate the yield (g/plant) per treatment replication. Finally, five of these plants were pooled, and their fresh biomass was immediately stored at −80 °C to determine the impact of the different N managements on purslane biochemical properties.

### 4.4. Determination of Nutrient Content

The analytical procedure that was followed to determine the nutrient concentration in both plant tissues and NS samples was consistent with the methodology employed by Voutsinos-Franztis et al. [90]. Particularly, the pulverized dried shoot and root samples were subjected to dry ashing at 550 °C for 8 h, and the resultant ash was then dissolved in 0.1 N HCl. In both plant extracts and NS samples, P and B were determined colorimetrically via Murphy and Riley [91] and azomethine [92] methods, while the nutrients K, Na, Ca, Mg, Fe, Zn, Cu, and Mn were assessed using an atomic absorption spectrophotometer (AA-7000, Shimadzu Co., Ltd., Tokyo, Japan). The NO3-N and NH4-N levels in NS samples were also determined colorimetrically by applying VCl3 reduction [93] and indophenol blue [94] methods, respectively. Additionally, the dried plant tissues were subjected to a hot water extraction [95] to determine Cl and NO_3_ concentration via mercury thiocyanate [96] and salicylic acid method [97], respectively. The reduced N (Nred) content was determined via the Kjeldahl method [98]. Subsequently, the values of NO_3_-N (Nmin) and reduced-N were then added to calculate the total-N (Ntotal) content of the dried plant tissues. All analytical measurements were performed in duplicate, with two technical replicates per sample.

### 4.5. Determination of Plant Biochemical Profile

To determine the impact of the different treatments on plant biochemical profile, the fresh plant tissue samples were freeze-dried and subjected to methanolic extraction as described by Karavidas et al. [99]. Ferric reducing antioxidant power (FRAP) [100] and Trolox equivalent antioxidant capacity (TEAC) [101] assays were employed to determine the antioxidant properties of purslane extracts, and the values were expressed in Trolox equivalents per gram of dry biomass. The total phenolic content of purslane was estimated using the Folin–Ciocalteu reagent method as described by Jan et al. [102], and the values were expressed in mg gallic acid equivalent per g of dry biomass. Finally, to assess the total flavonoid content, the aluminum chloride colorimetric method was employed as described by Safafar et al. [103], and the values were expressed in mg quercetin equivalent per g of dry biomass. All biochemical analyses were conducted in duplicate, with two technical replicates performed per sample.

### 4.6. Nutrient Uptake Concentrations

The uptake concentrations (UCs) of macro- (N, P, K, Ca, Mg, and Na) and micronutrients (B, Fe, Zn, Mn, and Cu) were estimated by considering the nutrient levels in either the NS (UCs-NS) or plant dry biomass (UC-DB) and the total volume of water consumed by plants [66]. Particularly, the UCs-DB were determined using Equation (1):

UC-DBx = (Cx × DBs + Cx × DBr)/(Vw × Arx)(1)

where “x” denotes the nutrient, “C” denotes the concentration of the “x” nutrient in dry biomass (in mg per g of dry biomass), “DBs-r” denotes for the weight (g) of dried biomass of shoot and root, respectively, “Vw” the total volume (L) of water consumed by plant, and “Arx” denotes for the atomic weight (g/mol) of “x” nutrient.

Accordingly, the UCs-NS were calculated using the following equation as suggested by Savvas et al. [104]:

UC-NSx = (Cex × Ve − Ctx × Vt)/[(Ve − Vt) × Arx](2)

where “x” denotes the nutrient, “Ce” denotes the concentration (mg/L) of “x” nutrient into the NS during the crop establishment, “Ve” denotes the volume (L) of NS during the crop establishment, “Ct” denotes the concentration (mg/L) of x nutrient at crop termination, “Vt” denotes for the volume (L) of NS at the crop termination, and “Arx” the molecular weight (g/mol) of x nutrient.

Both UCs were expressed in mM for macronutrients, while the obtained values of UCs were multiplied by 103 to express the results of micronutrient UCs in μM.

### 4.7. Nitrogen Use Efficiency 

The N utilization efficiency (NUtE), N uptake efficiency (NUpE), and N use efficiency (NUE) were determined according to Congreves et al. [105] using the following equations:

NUtE = Dry biomass (g/plant)/Plant N content (g/plant)(3)

NUpE = Plant N content (g/plant)/Total N applied via NS (g/plant)(4)

NUE = NUtE × NUpE(5)

### 4.8. Statistical Analysis

The recorded values of leaf number and leaf area of purslane plants per treatment replication were averaged. The obtained values along with those of the rest biomass characteristics, plant nutrient content, and biochemical profile were subjected to one-way ANOVA, with the main factor being the different treatments (Nr7, Nr14, N30, and N50, respectively). Additionally, the obtained UC values were subjected to factorial-ANOVA, with factor A the different N treatments and factor B the different estimation method (UC-DB vs. UC-NS). Once ANOVA analysis was significant at *p* ≤ 0.05, the treatment means were separated by applying Duncan’s multiple range test. In the current study, the statistical analysis was performed using the STATISTICA software package, version 12.0 for Windows (Tulsa, OK, USA).

## 5. Conclusions

The present study demonstrates that, although purslane exhibits substantial adaptability to suboptimal growing conditions, its yield and nutrient dynamics remain highly sensitive to both the rate and form of N supplied. A 30% partial substitution of NO_3_-N with Cl is recommended as an efficient fertilization strategy that optimizes both yield and product quality in hydroponically grown purslane. In contrast, further reductions in N supply or partial substitution with NH_4_-N are not advisable, as these practices have proved to offer limited quality benefits while significantly compromising plant growth and yield performance. Besides N management, further research should be conducted to address the optimal K/Ca ratio that allows for proper Ca utilization for the hydroponic production of purslane during the spring cultivation period. Discrepancies in UC between nutrient solution and plant tissue-based methods were minimal for most elements but seem to be highly influenced by the NS chemical properties and the applied analytical methodology. Despite these discrepancies, the estimation of plant UC values based on the NS analysis suggests a more practical method that generates more reliable data for refining nutrient solution formulations. Collectively, these findings offer a framework for fine-tuning nutrient delivery in closed-loop hydroponic systems and support the development of targeted fertilization strategies to maximize yield, nutrient efficiency, and product quality in purslane cultivation during spring conditions.

## Figures and Tables

**Table 1:** Impact of the different N management treatments on yield, leaf number (LN), leaf area (LA), shoot dry weight (SDW), aboveground biomass dry matter content (DMC), and root dry weight (RDW) of hydroponically grown purslane.

N Treatments	Yield	LN	LA	SDW	DMC	RDW
	(g Plant^−1^)	(N Plant^−1^)	(cm^2^ Plant^−1^)	(g Plant^−1^)	(%)	(g Plant^−1^)
Nr7	84.8 a	202 a	748 a	3.00 a	3.53 b	0.218 a
Nr14	68.5 c	168 b	600 c	2.60 b	3.80 a	0.188 b
N30	81.7 ab	204 a	737 ab	2.93 a	3.59 b	0.202 ab
N50	76.5 b	183 ab	660 bc	2.78 ab	3.64 b	0.203 ab
Statistical Significance	***	*	**	*	**	*

Mean values within the same column followed by different letters indicate statistically significant differences according to the Duncan multiple range test at *p* < 0.05. *, ** and *** denote significance at *p* < 0.05, *p* < 0.01, and *p* < 0.001, respectively. Nr7 denotes for 7% constitution of nitrate nitrogen (NO_3_-N) with ammonium nitrogen (NH_4_-N), Nr14 denotes for 14% constitution of nitrate nitrogen (NO_3_-N) with ammonium nitrogen (NH_4_-N), N30 denotes for compensating 30% of N inputs with Cl, and N50 denotes for compensating 50% of N inputs in the nutrient solution (NS) with 30% Cl and 20% S. Standard errors for each mean value are reported in Table S1.

**Table 2:** Impact of N management treatments on N reduced forms (Nred), NO_3_-N content (Nmin), total N content (Nred + Nmin), and Nred/Ntotal ratio in plant aboveground dry biomass and nitrate accumulation (NO_3_) in fresh produce.

N Treatments	Nred	Nmin	Ntotal	Nred/Ntotal	NO_3_
	(%)	(%)	(%)		(mg kg^−1^ FW)
Nr7	4.08 a	1.82 a	5.90 a	0.692 b	2877 a
Nr14	3.63 c	1.85 a	5.48 b	0.663 c	2902 a
N30	3.97 ab	1.62 b	5.59 b	0.710 ab	2588 b
N50	3.94 b	1.57 b	5.50 b	0.717 a	2585 b
Statistical significance	***	***	***	***	***

Mean values within the same column followed by different letters indicate statistically significant differences according to the Duncan multiple range test at *p* < 0.05. *** denotes significance at *p* < 0.001. Nr7 denotes for 7% constitution of nitrate nitrogen (NO_3_-N) with ammonium nitrogen (NH_4_-N), Nr14 denotes for 14% constitution of nitrate nitrogen (NO_3_-N) with ammonium nitrogen (NH_4_-N), N30 denotes for compensating 30% of N inputs with Cl, and N50 denotes for compensating 50% of N inputs in the nutrient solution (NS) with 30% Cl and 20% S. Standard errors for each mean value are reported in Table S2.

**Table 3:** Impact of N management treatments on macro- (P, K, Ca, Mg, Na, and Cl) and micronutrients (B, Fe, Mn, Zn, and Cu) concentrations on plant aboveground dry biomass.

Nutrient	Unit (D.W.)	Nr7	Nr14	N30	N50	Statistical Significance
P	mg/g	8.23 b	9.82 a	8.77 b	9.13 ab	*
K	mg/g	143	145	138	146	ns
Ca	mg/g	0.308	0.295	0.307	0.282	ns
Mg	mg/g	8.37	8.53	7.80	7.93	ns
Na	mg/g	3.00 b	3.93 a	2.88 b	3.46 ab	*
Cl	mg/g	12.08 c	12.29 c	24.79 b	26.38 a	***
B	μg/g	52.8	62.3	64.1	64.0	ns
Fe	μg/g	106.9 a	91.4 b	101.7 a	89.1 b	**
Zn	μg/g	232	230	237	246	ns
Mn	μg/g	194	194	195	181	ns
Cu	μg/g	18.7 b	21.7 a	20.0 ab	21.0 a	*

Mean values within the same column followed by different letters indicate statistically significant differences according to the Duncan multiple range test at *p* < 0.05. *, **, and *** denote significance at *p* < 0.05, *p* < 0.01, and *p* < 0.001, respectively. ns = non-significant. Nr7 denotes for 7% constitution of nitrate nitrogen (NO_3_-N) with ammonium nitrogen (NH_4_-N), Nr14 denotes for 14% constitution of nitrate nitrogen (NO_3_-N) with ammonium nitrogen (NH_4_-N), N30 denotes for compensating 30% of N inputs with Cl, and N50 denotes for compensating 50% of N inputs in the nutrient solution (NS) with 30% Cl and 20% S. Standard errors for each mean value are reported in Table S3.

**Table 4:** Impact of N management treatments on macro- (N, P, K, Ca, Mg, and Na) and micronutrients (B, Fe, Mn, Zn, and Cu) concentrations on plant root dry biomass.

Nutrient	Unit (D.W.)	Nr7	Nr14	N30	N50	Statistical Significance
N	mg/g	42.5	42.9	42.9	42.7	ns
P	mg/g	7.93	7.86	7.73	7.64	ns
K	mg/g	49.0	45.3	52.5	56.0	ns
Ca	mg/g	0.476	0.503	0.499	0.472	ns
Mg	mg/g	3.98	3.99	3.94	4.09	ns
Na	mg/g	0.463	0.517	0.488	0.550	ns
B	μg/g	60.5	62.2	65.1	68.4	ns
Fe	μg/g	1063	1196	1300	1293	ns
Zn	μg/g	646	588	643	689	ns
Mn	μg/g	136	130	157	165	ns
Cu	μg/g	54.5 b	65.4 a	54.1 b	60.2 ab	*

Mean values within the same column followed by different letters indicate statistically significant differences according to the Duncan multiple range test at *p* < 0.05. * denotes significance at *p* < 0.05. ns = non-significant. Nr7 denotes for 7% constitution of nitrate nitrogen (NO3-N) with ammonium nitrogen (NH_4_-N), Nr14 denotes for 14% constitution of nitrate nitrogen (NO3-N) with ammonium nitrogen (NH_4_-N), N30 denotes for compensating 30% of N inputs with Cl, and N50 denotes for compensating 50% of N inputs in the nutrient solution (NS) with 30% Cl and 20% S. Standard errors for each mean value are reported in Table S4.

**Table 5:** Impact of the uptake concentrations (UC) estimation method (UC-NS vs. UC-DB) and the N management treatments on macronutrient (N, P, K, Ca, Mg, Na, and Cl) UCs of hydroponic purslane.

Macronutrient UC (mM)								
Main Effects								
UC Method	N Treatments	N	P	K	Ca	Mg	Na	Cl
UC-NS		13.48 a	1.09 a	12.74 a	0.435 a	1.18 a	0.506 a	1.85
UC-DB		13.13 b	0.95 b	11.61 b	0.026 b	1.07 b	0.444 b	1.68
	Nr7	14.39 a	1.02	12.37	0.197	1.19	0.461	1.15 b
	Nr14	13.37 b	1.05	11.94	0.279	1.08	0.526	1.10 b
	N30	13.10 b	0.97	12.04	0.250	1.12	0.44	2.33 a
	N50	12.39 c	1.04	12.30	0.208	1.11	0.485	2.32 a
**Interactions**								
UC-NS	Nr7	14.42 a	1.11	12.57	0.365	1.23	0.493	1.19
	Nr14	14.43 a	1.10	12.57	0.534	1.09	0.536	1.17
	N30	12.95 b	1.00	12.66	0.472	1.20	0.485	2.45
	N50	12,37 b	1.14	13.12	0.392	1.21	0.516	2.41
UC-DB	Nr7	14.35 a	0.92	12.17	0.028	1.15	0.428	1.10
	Nr14	12.31 b	1.00	11.30	0.025	1.08	0.515	1.03
	N30	13.25 b	0.94	11.42	0.027	1.05	0.396	2.20
	N50	12.40 b	0.94	11.49	0.024	1.01	0.454	2.23
**Statistical significance**								
UC method		*	***	**	***	*	**	ns
N treatment		***	ns	ns	ns	ns	*	***
UC method* N treatment		**	ns	ns	ns	ns	ns	ns

Mean values within the same column followed by different letters indicate statistically significant differences according to the Duncan multiple range test at *p* < 0.05. *, ** and *** denote significance at *p* < 0.05, *p* < 0.01, and *p* < 0.001, respectively. ns = non-significant. Nr7 denotes for 7% constitution of nitrate nitrogen (NO3-N) with ammonium nitrogen (NH_4_-N), Nr14 denotes for 14% constitution of nitrate nitrogen (NO3-N) with ammonium nitrogen (NH_4_-N), N30 denotes for compensating 30% of N inputs with Cl, and N50 denotes for compensating 50% of N inputs in the nutrient solution (NS) with 30% Cl and 20% S. UC-NS and UC-DB denote the uptake concentrations (UCs) of nutrients based on the nutrient levels in the nutrient solution (NS) and the nutrient content in plant dry biomass (DB). Standard errors for each mean value are reported in Table S5.

**Table 6:** Impact of the uptake concentrations (UC) estimation method (UC-NS vs. UC-DB) and N management treatments on micronutrient (B, Fe, Zn, Mn, and Cu) UCs of hydroponic purslane.

Micronutrient UC (μM)						
Main Effect						
UC Method	N Treatment	Β	Fe	Zn	Mn	Cu
UC-NS		21.25 a	12.17 a	14.38 a	14.38 a	1.25
UC-DB		18.58 b	10.20 b	13.30 b	11.39 b	1.15
	Nr7	19.39	11.50	13.76	13.30	1.24
	Nr14	19.26	10.79	13.03	12.98	1.18
	N30	20.30	11.26	14.24	12.96	1.18
	N50	20.55	11.09	14.12	12.33	1.20
**Statistical significance**						
UC method		**	***	*	***	ns
N treatment		ns	ns	ns	ns	ns
UC Method * N treatment		ns	ns	ns	ns	ns

Mean values within the same column followed by different letters indicate statistically significant differences according to the Duncan multiple range test at *p* < 0.05. *, ** and *** denote significance at *p* < 0.05, *p* < 0.01 and *p* < 0.001, respectively. ns = non-significant. Nr7 denotes for 7% constitution of nitrate nitrogen (NO_3_-N) with ammonium nitrogen (NH_4_-N), Nr14 denotes for 14% constitution of nitrate nitrogen (NO_3_-N) with ammonium nitrogen (NH_4_-N), N30 denotes for compensating 30% of N inputs with Cl, and N50 denotes for compensating 50% of N inputs in the nutrient solution (NS) with 30% Cl and 20% S. UC-NS and UC-DB denotes for the uptake concentrations (UCs) of nutrients based on the nutrient levels in the nutrient solution (NS) and the nutrient content in plant dry biomass (DB). Standard errors for each mean value are reported in Table S6.

**Table 7:** Chemical composition of NSs among the different treatments.

Nutrient	Nr7	Nr14	N30	N50	Unit
EC	2.6	2.6	2.6	2.6	dS/m
pH	5.3	5.3	5.3	5.3	
NO^3−^	14	12.9	9.8	7	mM
K^+^	7.77	7.40	7.77	7.94	mM
Ca^2+^	4.58	4.36	4.58	4.68	mM
Mg^2+^	2.98	2.84	2.98	3.05	mM
SO_4_^2−^	4.24	4.79	4.24	5.98	mM
H_2_PO_4_^−^	1.4	1.4	1.4	1.4	mM
NH_4_^+^	1	2.1	1	0.5	mM
Fe	20	20	20	20	μM
Mn^2+^	9	9	9	9	μM
Zn^2+^	5	5	5	5	μM
B	30	30	30	30	μM
Cu^2+^	0.8	0.8	0.8	0.8	μM
Mo	0.5	0.5	0.5	0.5	μΜ
Cl^−^	0.4	0.4	4.6	3.9	μΜ
K^+^/(K^+^ + Ca^2+^ + Mg^2+^)	0.51	0.51	0.51	0.51	mol/mol
Ca^2+^/(K + Ca^2+^ + Mg^2+^)	0.3	0.3	0.3	0.3	mol/mol
Mg^2+^ (K^+^ + Ca^2+^ + Mg^2+^)	0.19	0.19	0.19	0.19	mol/mol
N/K^+^	1.93	2.03	1.39	0.94	mol/mol
NH_4_−N/Total−N	0.07	0.14	0.09	0.07	mol/mol

## Data Availability

The original contributions presented in this study are included in the article/Supplementary Materials.

## Supplementary Materials

The following supporting information can be downloaded at https://www.mdpi.com/article/10.3390/plants14142160/s1, Figure S1: Fluctuation of pH level during the growing season. Data points represent mean values ± SE. Nr7 denotes for 7% substitution of nitrate nitrogen (NO_3_-N) with ammonium nitrogen (NH_4_-N), Nr14 denotes for 14% substitution of nitrate nitrogen (NO_3_-N) with ammonium nitrogen (NH_4_-N), N30 denotes for compensating 30% of N inputs with Cl, and N50 denotes for compensating 50% of N inputs in the nutrient solution (NS) with 30% Cl and 20% S. Figure S2: Fluctuation of electrical conductivity (EC) level during the growing season. Data points represent mean values ± SE. Nr7 denotes for 7% substitution of nitrate nitrogen (NO3-N) with ammonium nitrogen (NH_4_-N), Nr14 denotes for 14% substitution of nitrate nitrogen (NO3-N) with ammonium nitrogen (NH_4_-N), N30 denotes for compensating 30% of N inputs with Cl, and N50 denotes for compensating 50% of N inputs in the nutrient solution (NS) with 30% Cl and 20% S. Table S1. Standard errors (S.E.) of the mean values reported in Table 1: Impact of the different N management treatments on yield, leaf number (LN), leaf area (LA), shoot dry weight (SDW), aboveground biomass dry matter content (DMC), and root dry weight (RDW) of hydroponically grown purslane. Table S2. Standard errors (S.E.) of the mean values reported in Table 2: Impact of N management treatments on N reduced forms (Nred), NO_3_-N content (Nmin), total N content (Nred + Nmin), and Nred/Ntotal ratio in plant aboveground dry biomass and nitrate accumulation (NO_3_) in fresh produce. Table S3. Standard errors (S.E.) of the mean values reported in Table 3: Impact of N management treatments on macro- (P, K, Ca, Mg, Na, and Cl) and micronutrients (B, Fe, Mn, Zn, and Cu) concentrations on plant aboveground dry biomass. Table S4. Standard errors (S.E.) of the mean values reported in Table 4: Impact of N management treatments on macro- (N, P, K, Ca, Mg, and Na) and micronutrients (B, Fe, Mn, Zn, and Cu) concentrations on plant root dry biomass. Table S5. Standard errors (S.E.) of the mean values reported in Table 5: Table 5. Impact of the uptake concentrations (UC) estimation method (UC-NS vs UC-DB) and the N management treatments on macronutrient (N, P, K, Ca, Mg, Na, and Cl) UCs of hydroponic purslane. Table S6. Standard errors (S.E.) of the mean values reported in Table 6: Impact of the uptake concentrations (UC) estimation method (UC-NS vs. UC-DB) and N management treatments on micronutrient (B, Fe, Zn, Mn, and Cu) UCs of hydroponic purslane.
