# Supplementary material for: Chloride as a Partial Nitrate Substitute in Hydroponics: Effects on Purslane Yield and Quality
Source: Plants (Basel). 2025 Jul 13;14(14):2160. doi: 10.3390/plants14142160 (PMC12299384; doi:10.3390/plants14142160)
Supplement: Supplementary file 1 [file plants-14-02160-s001.zip › plants-3739803-supplementary.pdf]

Figure S1. Fluctuation of pH level during the growing season

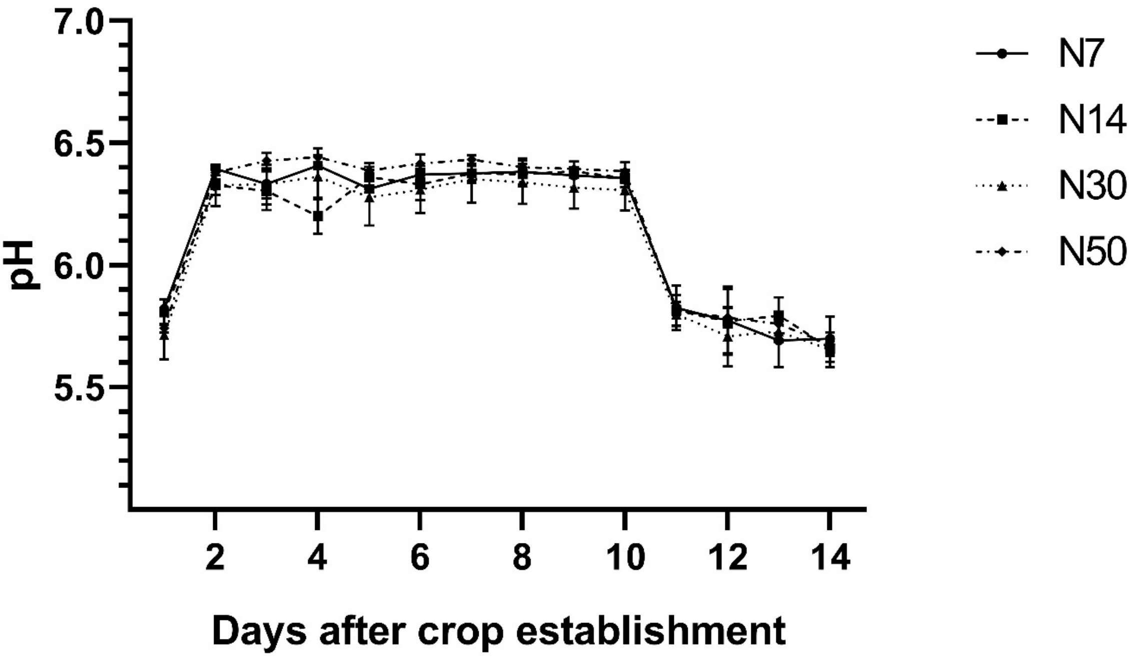

Data points represent mean values ± SE. Nr7 denotes for 7% constitution of nitrate nitrogen (NO_3-N) with ammonium nitrogen (NH_4-N), Nr14 denotes for 14% constitution of nitrate nitrogen (NO_3-N) with ammonium nitrogen (NH_4-N), N30 denotes for compensating 30% of N inputs with Cl, and N50 denotes for compensating 50% of N inputs in the nutrient solution (NS) with 30% Cl and 20% S.

It has been demonstrated that the pH level remained relatively constant throughout the cultivation period. The pH values exhibited no statistically significant differences among the N treatments.

Figure S2. Fluctuation of electrical conductivity (EC) level during the growing season

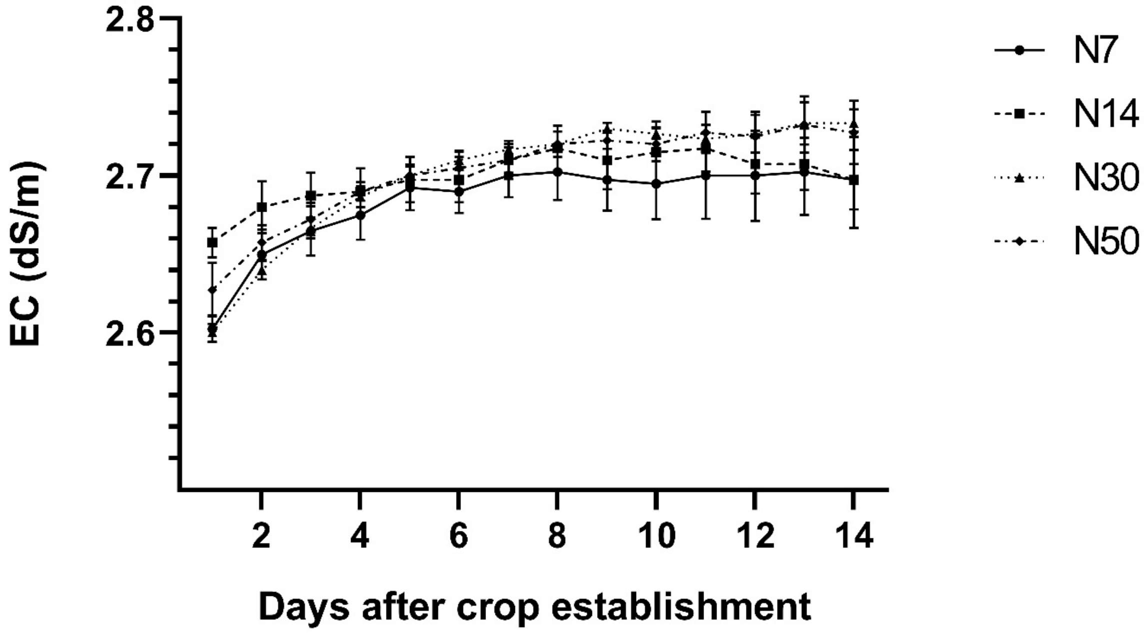

Data points represent mean values ± SE. Nr7 denotes for 7% constitution of nitrate nitrogen (NO_3-N) with ammonium nitrogen (NH_4-N), Nr14 denotes for 14% constitution of nitrate nitrogen (NO_3-N) with ammonium nitrogen (NH_4-N), N30 denotes for compensating 30% of N inputs with Cl, and N50 denotes for compensating 50% of N inputs in the nutrient solution (NS) with 30% Cl and 20% S.

Electrical conductivity (EC) values exhibited no significant differences between the N treatments. A marginal increase in electrical conductivity (EC) has been observed in each tank during the cultivation period, with the EC levels remaining well below the acceptable limits.

Table S1. Standard errors (S.E.) of the mean values reported on Table 1: Impact of the different N management treatments on yield, leaf number (LN), leaf area (LA), shoot dry weight (SDW), aboveground biomass dry matter content (DMC) and root dry weight (RDW) of hydroponically grown purslane.

Biomass characteristics S.E.						
Main effects						
**N treatments**	**Yield** (g plant^{-1})	**LN** (N plant^{-1})	**LA** (cm^{2}plant^{-1})	**SDW** (g plant^{-1})	**DMC** (%)	**RDW** (g plant^{-1})
Nr7	1.934	5.687	15.429	0.100	0.054	0.002
Nr14	0.785	2.553	19.648	0.033	0.033	0.004
N30	2.213	8.238	36.671	0.072	0.055	0.006
N50	2.074	9.027	20.858	0.085	0.040	0.008

Table S2. Standard errors (S.E.) of the mean values reported on Table 2: Impact of N management treatments on N reduced forms (Nred), NO_3-N content (Nmin), total N content (Nred + Nmin) and Nred:Ntotal ratio in plant aboveground dry biomass and nitrate accumulation (NO_3) in fresh produce.

Plant nitrogen profile S.E.					
Main effects					
**N treatments**	**Nred** (%)	**Nmin** (%)	**Ntotal** (%)	**Nred/Ntotal**	**NO_3** (mg kg^{-1} FW)
Nr7	0.045	0.053	0.044	0.008	52.584
Nr14	0.029	0.056	0.068	0.007	61.173
N30	0.044	0.045	0.038	0.007	56.390
N50	0.045	0.051	0.059	0.008	86.828

Table S3. Standard errors (S.E.) of the mean values reported on Table 3: Impact of N management treatments on macro- (P, K, Ca, Mg, Na & Cl) and micronutri-ents (B, Fe, Mn, Zn & Cu) concentrations on plant aboveground dry biomass.

**Aboveground biomass nuntrient profile S.E.**					
Main effects					
**Nutrient**	**Unit (D.W.)**	**Nr7**	**Nr14**	**N30**	**N50**
P	mg/g	0.287	0.482	0.277	0.121
K	mg/g	3.161	5.053	6.135	2.619
Ca	mg/g	0.011	0.015	0.018	0.008
Mg	mg/g	0.345	0.335	0.306	0.313
Na	mg/g	0.136	0.307	0.199	0.187
Cl	mg/g	0.482	0.373	0.626	0.997
B	µg/g	2.826	3.148	4.655	3.304
Fe	µg/g	4.330	2.135	2.782	2.814
Zn	µg/g	6.177	10.148	7.486	9.513
Mn	µg/g	4.875	7.598	7.870	3.620
Cu	µg/g	0.572	0.880	0.529	0.568

Table S4. Standard errors (S.E.) of the mean values reported on Table 4: Impact of N management treatments on macro- (N, P, K, Ca, Mg & Na) and micronutrients (B, Fe, Mn, Zn & Cu) concentrations on plant root dry biomass.

**Root nuntrient profile S.E.**					
Main effects					
**Nutrient**	**Unit (D.W.)**	**Nr7**	**Nr14**	**N30**	**N50**
N	mg/g	0.480	0.741	0.400	0.813
P	mg/g	0.713	0.368	0.728	0.766
K	mg/g	3.416	2.906	3.304	2.160
Ca	mg/g	0.025	0.056	0.024	0.033
Mg	mg/g	0.151	0.068	0.036	0.251
Na	mg/g	0.031	0.033	0.055	0.035
B	µg/g	5.695	11.885	7.315	10.505
Fe	µg/g	102.796	132.895	199.706	138.070
Zn	µg/g	115.272	57.561	11.375	47.819
Mn	µg/g	10.813	8.959	5.897	10.184
Cu	µg/g	1.641	1.575	1.394	3.303

Table S5. Standard errors (S.E.) of the mean values reported on Table 5: Table 5. Impact of the uptake concentrations (UC) estimation method (UC-NS vs UC-DB) and the N management treatments on macronutrient (N, P, K, Ca, Mg, Na & Cl) UCs of hydroponic purslane.

**Macronutrient UC (mM) S.E.**								
Main effects								
UC method	N treatments	N	P	K	Ca	Mg	Na	Cl
UC-NS		0.259	0.024	0.237	0.034	0.031	0.011	0.182
UC- DB		0.270	0.021	0.210	0.001	0.032	0.017	0.153
	Nr7	0.132	0.045	0.254	0.067	0.042	0.017	0.093
	Nr14	0.480	0.038	0.321	0.126	0.041	0.025	0.081
	N30	0.278	0.029	0.531	0.086	0.048	0.023	0.074
	N50	0.171	0.047	0.357	0.077	0.059	0.020	0.089
Interactions								
UC-NS	Nr7	0.150	0.039	0.388	0.042	0.046	0.016	0.198
	Nr14	0.152	0.029	0.138	0.119	0.081	0.029	0.167
	N30	0.272	0.039	0.856	0.042	0.052	0.021	0.099
	N50	0.202	0.049	0.157	0.069	0.074	0.023	0.173
UC-DB	Nr7	0.240	0.043	0.351	0.001	0.072	0.018	0.016
	Nr14	0.094	0.063	0.300	0.001	0.040	0.046	0.014
	N30	0.522	0.042	0.574	0.002	0.064	0.026	0.073
	N50	0.309	0.028	0.355	0.001	0.062	0.026	0.039

Table S6. Standard errors (S.E.) of the mean values reported on Table 6: Impact of the uptake concentrations (UC) estimation method (UC-NS vs UC-DB) and N management treatments on micronutrient (B, Fe, Zn, Mn & Cu) UCs of hydroponic purslane.

**Micronutrient UC (μM) S.E.**						
Main effect						
UC method	N treatment	B	Fe	Zn	Mn	Cu
UC-NS		0.592	0.333	0.349	0.203	0.053
UC-DB		0.547	0.281	0.234	0.317	0.019
	Nr7	0.960	0.488	0.453	0.582	0.072
	Nr14	0.697	0.716	0.494	0.899	0.030
	N30	0.963	0.525	0.466	0.626	0.074
	N50	1.056	0.640	0.403	0.710	0.042
